# Diseases of Old Age in Two Paintings by Rembrandt

**DOI:** 10.5041/RMMJ.10227

**Published:** 2015-10-26

**Authors:** George M. Weisz, William R. Albury

**Affiliations:** 1 Adjunct Senior Lecturer, School of Humanities and Languages (Program in History of Medicine), University of New South Wales, Sydney, Australia, and School of Humanities, University of New England, Armidale, New South Wales, Australia; 2 Adjunct Professor, School of Humanities, University of New England, Armidale, New South Wales, Australia

**Keywords:** Diseases in art, gerontology, joint diseases, Rembrandt van Rijn

## Abstract

Two paintings of older men by Rembrandt (1609–1669) are examined to demonstrate that historical attitudes toward diseases of old age and the ageing person’s response to illness can be investigated in paintings. The works selected are of different genres and date from different stages of Rembrandt’s own life, one from his youth and one from his old age. Both paintings show figures who have joint pathologies typically associated with the ageing process, the first involving the subject’s foot and the second involving the subject’s hand. Despite the sometimes painful nature of these conditions, the subjects are shown accommodating their illnesses while maintaining both their intellectual and social engagement and their emotional composure. Although the seventeenth century offered older people very little effective medical treatment in comparison with what is presently available, these paintings nevertheless present a view of illness as a subsidiary rather than a dominant feature of old age.

## INTRODUCTION

The Dutch artist Rembrandt van Rijn (1606–1669) produced many memorable depictions of older persons. Indeed, more than half his extant paintings show figures who are ageing,^1^ not to mention his drawings and etchings. Like other Dutch and Flemish artists of his time—such as Peter-Paul Rubens (1577–1640), who was also interested in ageing people as subjects for his paintings—Rembrandt tended to focus more on the realistic portrayal of old age than on idealized or allegorical treatments.^2^ This is not to say, however, that one can approach his figures as if they were exact replicas of the persons shown, since like all artists Rembrandt developed his own individual style of representation which could adjust, exaggerate, or downplay features of a person’s appearance for aesthetic purposes.^3^

It has been suggested that paintings of older adults, by Rembrandt and other artists, can give modern gerontologists insight into the ways in which ageing was viewed in earlier times, with regard to such issues as the subjects’ engagement with or detachment from the world, their portrayal as embodiments of wisdom or folly, and the extent of their interaction with people of other generations.^4^ In addition to these considerations it is also of interest to know how the diseases of old age were viewed in previous centuries. The present study examines two works by Rembrandt to demonstrate that earlier attitudes toward the diseases characteristic of ageing can be investigated in paintings.

Rembrandt, undoubtedly the greatest artist of the Netherlands, was one of the outstanding painters of the seventeenth century. He began his career in Leiden, the city of his birth, and later moved to Amsterdam where he achieved his greatest successes. Despite his fame, his family life was plagued by tragedies, with the loss of most of his closest loved ones: his wife, a subsequent *de facto* partner, two daughters, and two sons. Ultimately he was declared bankrupt, abandoned by the Amsterdam painters’ guild and by society generally, and forced to sell his home, furniture, and art works to pay his debts. Today, however, his masterpieces are housed in an Amsterdam museum which carries his name and in many other galleries around the world.^5^

Two paintings by Rembrandt in the National Gallery of Victoria, Melbourne, Australia, will be briefly considered. One of them is a narrative scene showing two old men, dating from the artist’s youth. The other is a portrait of an ageing man completed in Rembrandt’s final years—a time when we might expect him to have been disillusioned with the world, in view of his own unhappy experiences. This selection allows us to examine two different genres of painting from two different periods in Rembrandt’s life.

In what follows we shall give a description of each painting and identify the pathologies depicted.

Then we shall discuss the significance of these two paintings for the conception of illness and old age which they present. It would be foolhardy to generalize about Rembrandt’s attitude toward ageing and illness from the inspection of two paintings only, and that is not the purpose of the present exercise. Rather, our intention is to show that in these two specific pictures one can see a representation of ageing in which physical illnesses typically associated with the later stages of life are accommodated by such simple expedients as resting an affected limb. The joint pathologies shown in these two works would have caused the subjects pain at times, but the individuals depicted have lost neither their intellectual and social engagement with the world nor their emotional composure.

## THE PAINTINGS

**Figure 1:**
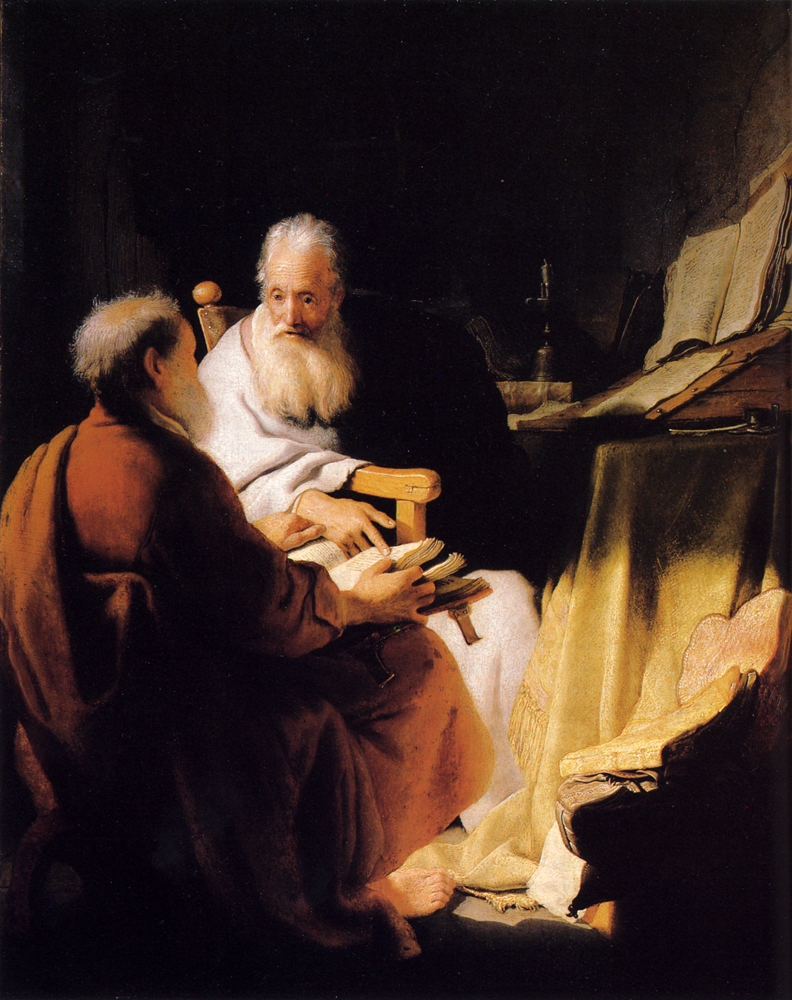
Rembrandt Harmensz. van Rijn, Dutch 1606–1669, *Two Old Men Disputing* (1628) Oil on wood panel, 72.4 × 59.7 cm, Felton Bequest, 1936; with permission of the National Gallery of Victoria, Melbourne, Australia.

**Figure 2:**
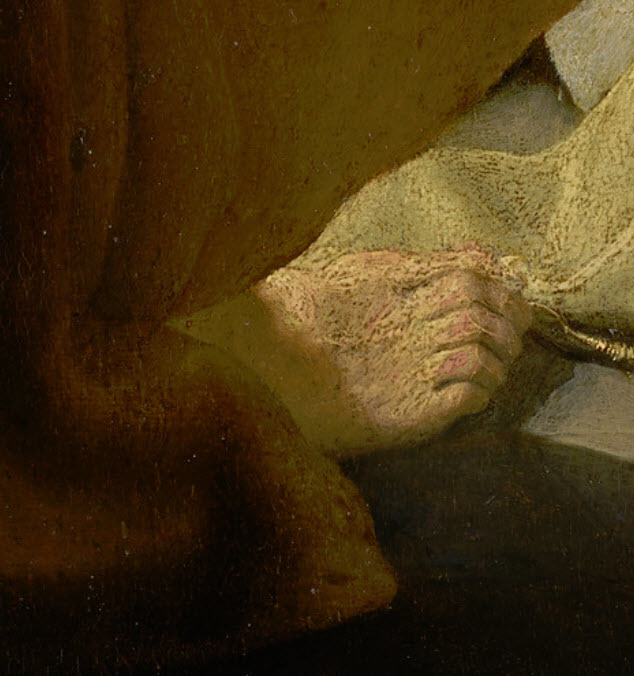
Detail of Figure 1, with permission of the National Gallery of Victoria, Melbourne, Australia

*Two Old Men Disputing* (Figure 1) was painted by Rembrandt in 1628, when he was aged 22 and still living in Leiden.^6^ It is not clear who the two old men are supposed to represent; suggestions have included the Hebrew prophets Elijah and Elisha, the Greek philosophers Hippocrates and Democritus, and the Christian apostles Peter (with his back toward the viewer) and Paul (facing the viewer). The currently accepted view favors this last interpretation, even though characteristic symbols such as Peter’s keys and Paul’s sword are not shown. Much of the painting is dimly lit, but a bright light falling between the two men illuminates Paul’s face, Peter’s right foot, and both men’s hands. The hands that are visible are normal, but Peter’s foot (Figure 2) is typical of an old man with deviated big toe and bunion (hallux valgus) forcing the rest of the toes into a hammer deformity at the proximal interphalangeal joint levels.

**Figure 3:**
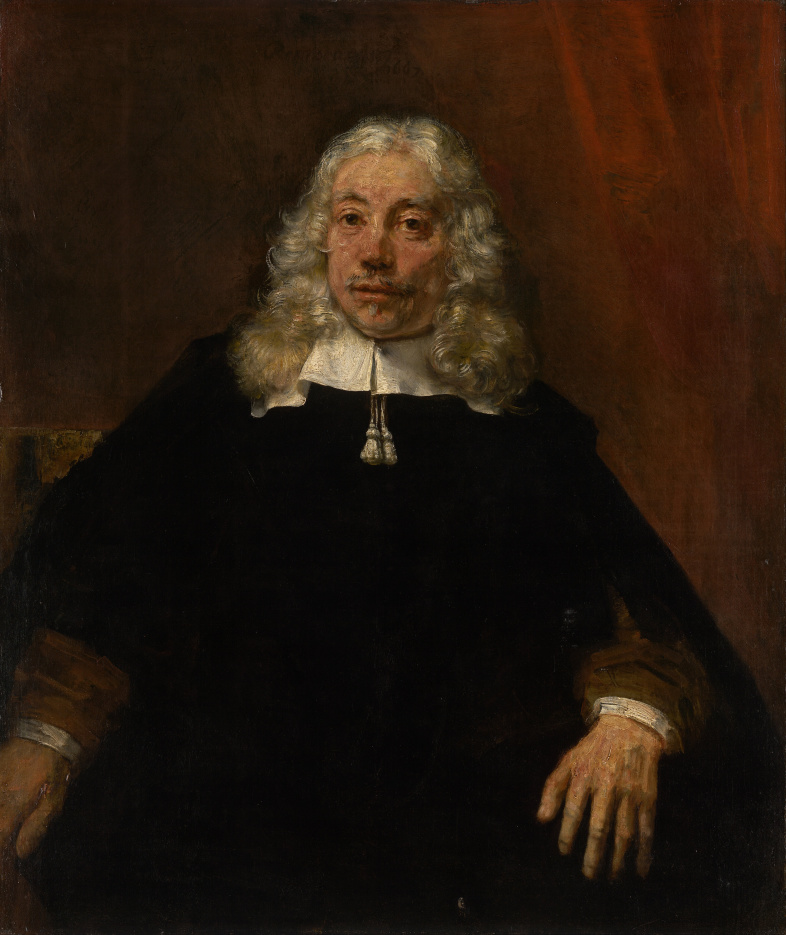
Rembrandt Harmensz. van Rijn, Dutch 1606–1669, *Portrait of a White-haired Man* (1667) Oil on canvas, 108.9 × 92.7 cm, Felton Bequest, 1951; with permission of the National Gallery of Victoria, Melbourne, Australia.

**Figure 4:**
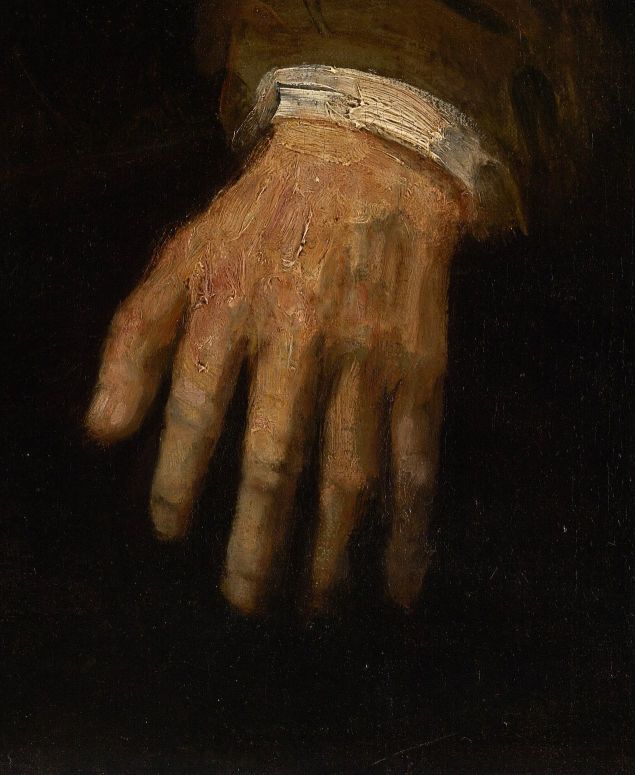
Detail of Figure 3, with permission of the National Gallery of Victoria, Melbourne, Australia

*Portrait of a White-haired Man* (Figure 3), from 1667, was one of Rembrandt’s last works.^6^ The identity of the sitter is unknown, but he is younger than the two men in the previous painting. His face shows effects of the ageing process but not to the extreme extent so well demonstrated in the 1628 depiction of the apostle Paul. There are only slight wrinkles around the eyes and mouth; the moustache and facial hair are partially whitish, but mixed with blond and with darker, brownish areas. The hair is abundant, and, to judge from the top of the scalp where it is parted, it does not seem to be a wig. The sitter would therefore probably be a man of middle age by modern standards; but he would have been an aged person by the standards of his own time and society, in which a male who survived to young adulthood could be expected to live only until about 60.^7^ His right hand is not fully visible, but anatomical anomalies seen in his left hand (Figure 4) include the swelling of the proximal interphalangeal joints in all five fingers and the swelling and flexion of the distal interphalangeal joints in the index and middle finger. The thumb has a prominence at the metacarpo-phalangeal joint level. All fingers are thicker than normal, not uniform but irregular, unlike a normal hand or a fat hand. These features appear to be the late result of a previous inflammatory arthritis.

Rembrandt was a skilled draughtsman, so we can eliminate any likelihood that these pathological features shown in his subjects’ hands or feet were the result of the artist attempting to represent a normal limb but failing to depict it accurately. Nor are these features likely to be stylistic embellishments on Rembrandt’s part. As previously noted, artists will often distort an anatomical element in a painting or sculpture for aesthetic rather than representational reasons.^3^ In Rembrandt’s paintings, however, we find many instances throughout his career of normal hands and feet depicted on older subjects—for example, the normal hands shown in *Two Old Men Disputing*. So there is no evidence that the anatomical anomalies in the hand of the white-haired man or the foot of Peter were typical stylistic features of Rembrandt’s work.

## DISCUSSION

Both of the pathologies described above are associated with the ageing process, and both would cause discomfort when the limb in question was used. Walking on the deformed foot shown in *Two Old Men Disputing* would be painful, but as the figure is seated he is not putting pressure on it. Similarly, the joint disease shown in *Portrait of a White-haired Man* would cause the subject some degree of pain when he moved his hand, although the intensity of the pain might vary over time as the inflammation subsided or flared up again. An English contemporary with a similar condition, the parliamentarian Sir John Bramston (1611–1700), described the pain of an acute attack as being so severe that he was “unable to open or shut one hand without the help of the other.”^8^ The white-haired man’s hand is not acutely inflamed, however, so his pain when moving it would be mild to moderate. He sits with his hand relaxed, and his face reveals no evident suffering.

Another characteristic of both the pathologies shown in these two paintings is that they are not immediately conspicuous, but they become evident once the viewer’s attention is drawn to them. This subdued presentation of illness is significant, because it suggests that Rembrandt probably did not intend to make his subjects’ diseases a defining feature of their identity as ageing persons. Nevertheless, the pathologies are not concealed. Both Peter’s foot and the white-haired man’s hand are positioned in the foreground of their respective paintings and are well lit, standing out against darker surroundings. In these pictures the diseases of ageing are subordinated to other elements of the subjects’ personalities—they are a subsidiary rather than a dominant aspect of old age.

There seems to be no suggestion of depression or withdrawal from life in Rembrandt’s subjects. In *Two Old Men Disputing*, to judge from the face of Paul, the interlocutors are entirely absorbed in their discussion. But since this early painting was completed in Rembrandt’s youth, the question of the subject’s emotional state is of greater interest in connection with the *Portrait of a White-haired Man*, painted at a time when Rembrandt himself was coping with age, illness, and disappointment. There is, of course, no necessary connection between an artist’s personal circumstances and the emotional tone of his or her work. But since it is entirely possible that figures depicted by an unhappy artist might in some cases reflect the artist’s mood (especially in view of Leonardo da Vinci’s dictum that every painter paints himself),^9^ the question seems worthy of consideration in connection with Rembrandt’s white-haired man.

It has been suggested that evidence of depression (as well as the ageing process and various pathologies) can be seen in Rembrandt’s self-portraits from this period,^10^ and we know from his biography that he was often withdrawn in later life and would go for months without working.^10^,^11^ The extent to which Rembrandt actually suffered from depression is of course a somewhat speculative question,^2^,^12^ and one that is not critical to the present study. But if he did experience darker moods in his later years then he avoided projecting them onto the subject of his *Portrait*. The white-haired man is well dressed and well groomed. He is quiet, but his eyes are attentive and focused on the viewer, maintaining his social engagement. His face as a whole, although showing signs of age, is composed and alert.

## CONCLUSION

Although there are numerous depictions of older people in art works which portray the subject severely incapacitated by illness, many others, such as those we have examined here, represent ageing individuals who seem to be coping successfully with less serious pathological conditions.^1^ Such works show that even with the limited effectiveness of medical treatment in past centuries, as compared with what is available at present, the process of ageing was not necessarily regarded in earlier times as being dominated by illness. Despite the traditional characterization of the aged person as a figure of misery beset by diseases of every kind,^13^ many early modern artists produced works which conveyed “an overall impression of the dignity of old age despite infirmities.”^14^

## References

[1] McKee PL, Kauppinen H (1976). The Art of Aging: A Celebration of Old Age in the History of Western Art.

[2] Marcus E-L, Clarfield AM (2002). Rembrandt’s late self-portraits: psychological and medical aspects. Int J Aging Hum Dev.

[3] Philippot P, Appelboom T (1987). Stylistic and Documentary Understanding of Fine Arts. Art, History and Antiquity of Rheumatic Diseases.

[4] Kauppinen H, McKee P (1988). Old age, painting, and gerontology. J Aesth Ed.

[5] Clark K (1978). An Introduction to Rembrandt.

[6] Blankert A, Broos B, van der Wetering EJ (1997). Rembrandt: A Genius and his Impact.

[7] Johansson SR (2010). Medics, monarchs and mortality, 1600-1800: origins of the knowledge-driven health transition in Europe. University of Oxford Discussion Papers in Economic and Social History.

[8] Bramston J (1845). The Autobiography of Sir John Bramston.

[9] Kemp N, Clough CH (1976). ‘Ogni Dipintore Dipinge Se’: A Neoplatonic Echo in Leonardo’s Art Theory?. Cultural Aspects of the Italian Renaissance.

[10] Espinel CH (1999). Depression, physical illness, and the faces of Rembrandt. Lancet.

[11] Espinel CH (1997). A medical evaluation of Rembrandt. His self-portrait: ageing, disease, and the language of the skin. Lancet.

[12] Rothenberg A (1999). Depression, physical illness, and the faces of Rembrandt. Lancet.

[b13-rmmj-6-4-e0042] 13Juvenal. Satire 10, lines 218–19

[14] Weisz GM, Albury WR (2015). The hand in art: an old painting showing the osteoarthritis of old age. J Hand Surg.

